# Omega 3 supplementation reduces C-reactive protein, prostaglandin E_2_ and the granulocyte/lymphocyte ratio in heavy smokers: An open-label randomized crossover trial

**DOI:** 10.3389/fnut.2022.1051418

**Published:** 2022-12-01

**Authors:** Ingrid Elisia, Michelle Yeung, Sara Kowalski, Jennifer Wong, Hossein Rafiei, Roger A. Dyer, Sukhinder Atkar-Khattra, Stephen Lam, Gerald Krystal

**Affiliations:** ^1^The Terry Fox Laboratory, BC Cancer Research Centre, Vancouver, BC, Canada; ^2^Analytical Core for Metabolomics and Nutrition, BC Children's Hospital Research Institute, Vancouver, BC, Canada; ^3^Department of Integrative Oncology, British Columbia Cancer Research Institute, Vancouver, BC, Canada

**Keywords:** smokers, EPA, DHA, CRP, PGE_2_, blood cells

## Abstract

**Objectives:**

Given the current controversy concerning the efficacy of omega 3 supplements at reducing inflammation, we evaluated the safety and efficacy of omega 3 on reducing inflammation in people with a 6-year lung cancer risk >1.5% and a C reactive protein (CRP) level >2 mg/L in a phase IIa cross-over study.

**Materials and methods:**

Forty-nine healthy participants ages 55 to 80, who were still smoking or had smoked in the past with ≥30 pack-years smoking history, living in British Columbia, Canada, were randomized in an open-label trial to receive 2.4 g eicosapentaenoic acid (EPA) + 1.2 g docosahexaenoic acid (DHA)/day for 6 months followed by observation for 6 months or observation for 6 months first and then active treatment for the next 6 months. Blood samples were collected over 1 year for measurement of plasma CRP, plasma and red blood cell (RBC) membrane levels of EPA, DHA and other fatty acids, Prostaglandin E_2_ (PGE_2_), Leukotriene B_4_ (LTB_4_) and an inflammatory marker panel.

**Results:**

Twenty one participants who began the trial within the active arm completed the trial while 20 participants who started in the control arm completed the study. Taking omega 3 resulted in a significant decrease in plasma CRP and PGE_2_ but not LTB_4_ levels. Importantly, the effect size for the primary outcome, CRP values, at the end of the intervention relative to baseline was medium (Cohen's *d* = 0.56). DHA, but not EPA levels in RBC membranes inversely correlated with PGE_2_ levels. Omega 3 also led to a significant reduction in granulocytes and an increase in lymphocytes. These high-dose omega 3 supplements were well tolerated, with only minor gastrointestinal symptoms in a subset of participants.

**Conclusion:**

Omega 3 fatty acids taken at 3.6 g/day significantly reduce systemic inflammation with negligible adverse health effects in people who smoke or have smoked and are at high risk of lung cancer.

ClinicalTrials.gov, NCT number: NCT03936621.

## Introduction

Globally, lung cancer is the leading cause of cancer mortality in both men and women with over 1.8 million deaths/year (1). Importantly, tobacco smoking is the leading risk factor for developing lung cancer. Targeted screening of high-risk individuals between the age of 50/55 to 74 who have smoked or are still smoking have been shown to reduce lung cancer mortality (2, 3). The ability to identify individuals at high risk for lung cancer using risk factors or risk prediction models (4) provides the opportunity to evaluate chemoprevention in a screening population to prevent lung cancer. Chemoprevention refers to the use of dietary or pharmaceutical interventions to slow or reverse the progression of premalignancy to invasive cancer (5, 6). Despite decades of pre-clinical and clinical research, safe and low cost chemopreventive agents for lung cancer have not been identified (6).

Chronic inflammation has been shown to be a major player in lung cancer (7–10), and a number of studies have been carried out to correlate inflammatory biomarkers such as C-reactive protein (CRP), with the subsequent development of lung cancer (11). Recently, a large randomized double-blind trial involving 10,061 patients with previous myocardial infarction and a CRP level of ≥2 mg/L suggested anti-inflammatory therapy with canakinumab, a monoclonal antibody targeting interleukin-1, may be effective in reducing the incidence and mortality of lung cancer in addition to reducing non-fatal myocardial infarction, non-fatal stroke, or cardiovascular death (12, 13). In the secondary analysis, canakinumab treatment was associated with a dose-dependent reduction in CRP from 26 to 41% and of interleukin 6 from 25 to 43% (12). However, Canakinumab was associated with a higher incidence of fatal infection than was placebo and is costly.

Ample preclinical data suggest that the arachidonic acid—COX-2/prostaglandin E_2_ (PGE_2_) signaling pathway plays a pivotal role in promoting malignancy and that the inhibition of COX-2 and PGE_2_ synthesis suppresses lung tumorigenesis (14, 15). Studies in our laboratory with a mouse model of lung cancer have shown that fish oil, rich in eicosapentaenoic acid (EPA) and docosahexaenoic acid (DHA), was highly effective at preventing the tobacco smoke carcinogen, nicotine-derived nitrosamine ketone (NNK), from inducing lung nodules, and this was associated with a significant reduction in inflammatory cytokines and the lipid mediator, PGE_2_ (16, 17). The impact of omega 3 fatty acids (FAs) on PGE_2_ is of particular interest since EPA and DHA have been shown to reduce the conversion of arachidonic acid (AA), *via* cyclooxygenase 2 (COX2), to PGE_2_. These data support the antineoplastic effect of COX-2 inhibitors and provide the rationale for evaluating omega 3 FAs for their potential as chemoprevention agents for lung cancer. However, while there have been many mouse and human *in vitro* studies (18, 19), as well as *in vivo* mouse studies (20), that demonstrate omega 3 fatty acids reduce inflammation, their efficacy in humans remains controversial (21). Specifically, some human studies have shown a significant reduction in inflammatory markers (22–24), while others have shown no benefit at all (25, 26). Moreover, very few clinical trials have been carried out specifically to evaluate the safety and efficacy of omega 3 supplementation on the inflammatory status of people who smoke or who have smoked heavily (22, 23). We thus undertook a randomized cross-over clinical trial with high-risk individuals participating in a lung cancer screening study for people who had smoked or were still smoking with plasma CRP >2 mg/L to determine the effect of EPA + DHA natural product supplements on the levels of inflammatory markers.

## Materials and methods

### Study design and participants

People between the ages of 55 and 80 who were living in British Columbia and were still smoking or had smoked in the past with ≥30 pack-years smoking history, or a PLCOm2012 6-year lung cancer risk >1.5% in the International Lung Screening Trial (27) were invited to participate in the study. Inclusion criteria were: plasma C-reactive protein (CRP) > 2 mg/L, normal organ function, no evidence of lung cancer on low-dose computed tomography of the chest; not currently taking non-steroidal anti-inflammatory drugs, and not taking natural health products containing omega 3 FAs. Women of childbearing potential were required to document a negative pregnancy test prior to enrolment and agreed to use an acceptable form of birth control for the duration of the study. Exclusion criteria were: history of malignancy with the exception of non-melanomatous skin cancer, or uncontrolled intercurrent illness such as infection in the past 6 months, ongoing infection or chronic obstructive pulmonary disease or asthma requiring inhaled or oral corticosteroids.

**Figure 1:**
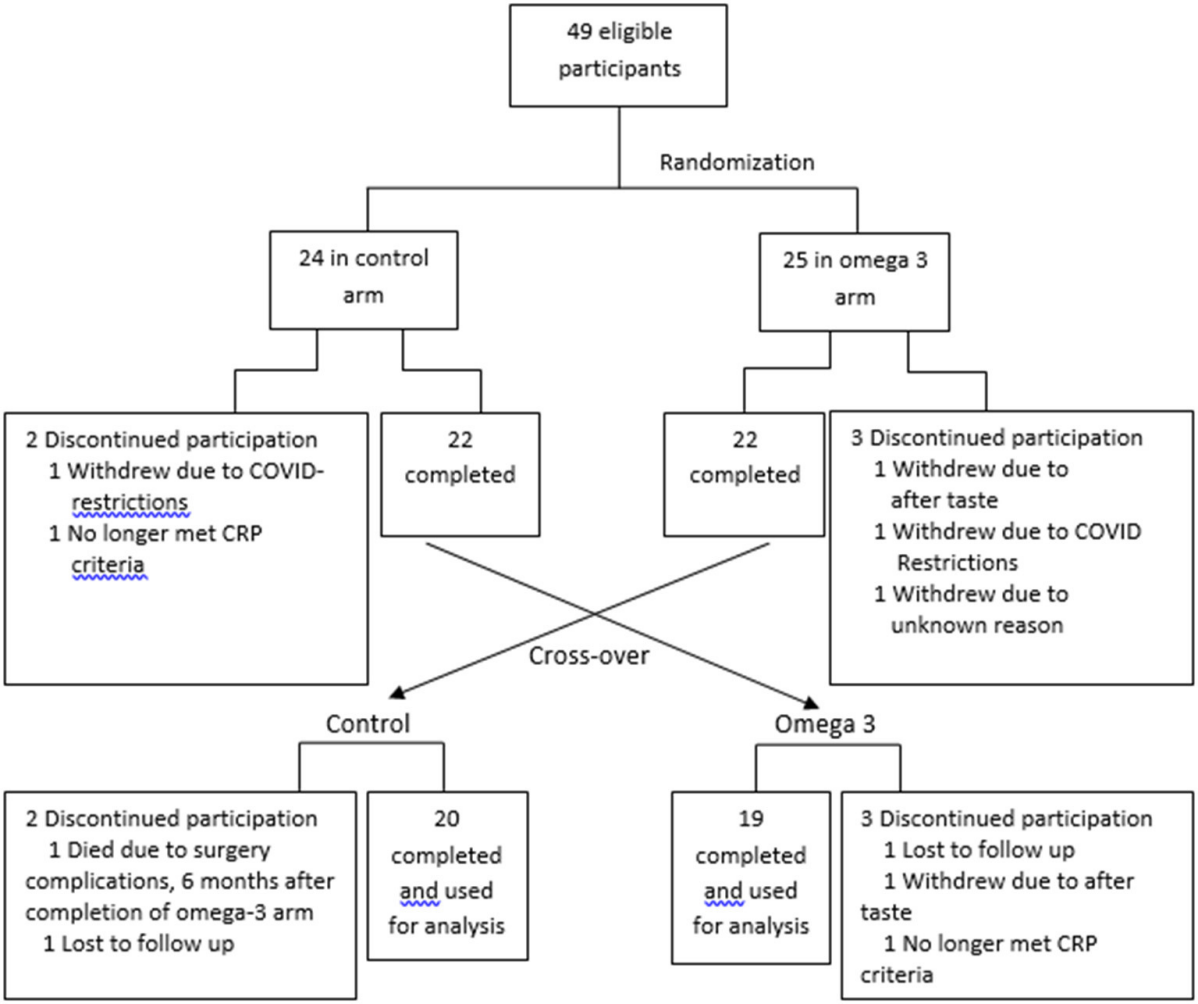
Study schema.

Between August 2019 and February 2020, subjects were randomly assigned 1:1 in an open-label, counterbalanced trial to take 4 capsules of Webber Triple strength omega 3 FAs)/day for the first 6 months or no treatment for 6 months (Figure 1). Informed consent was obtained prior to participation in the study. The study was approved by the Clinical Research Ethics board of the University of British Columbia and BC Cancer (H19-00221). To randomize the participants, a random numerical code was generated to assign participants to either active treatment or control. Each code was placed into a sealed envelope, and the envelopes were opened in numerical order by each participant that was deemed eligible to be in the study and after the consent form was signed. The project manager assigned the randomization and guarded the envelopes. The envelope was given to the study coordinator prior to the study participants' appointment.

Each capsule contained 600 mg eicosapentaenoic acid (EPA) + 300 mg docosahexaenoic acid (DHA); total 2.4 g EPA + 1.2 g DHA/day. The capsules were provided to the participants in white opaque bottles. They were blinded to the brand and the amount of EPA and DHA. At the end of 6-months, participants taking omega 3 were monitored off treatment for another 6 months to assess the kinetics of omega 3 FA clearance. Those who were not taking omega 3 in the first 6 months began taking them for the next 6 months. This randomized cross-over study was used so that each subject served as his or her own control. The last participants completed the trial in 2021.

### Compliance and adverse event monitoring

Compliance was assessed by capsule count and monitored using a diary, which was reviewed during a telephone call at month 1 and 7 or during an in person visit at months 3, 6, 9, and 12. Adverse events were classified and graded using NCI Common Terminology Criteria for Adverse Events version 5.0 (CTCAE v5.0) with the maximum grade per subject and event type recorded across the duration of intervention.

### Blood sample processing

The participants were required to fast overnight before each blood draw and blood was taken between 8 am and 10 am to avoid possible differences in cytokine expression due to diurnal rhythms (28). Two 10 mL EDTA Vacutainer tubes (cat. No. 366643, BD, Mississauga, ON) and one 6 mL SST Gold top tube (for serum) were collected by a trained phlebotomist at BC Cancer.

A 1 mL aliquot of the blood collected in a 10 ml EDTA tubes was centrifuged at 8,000×g for 5 min at 4°C. The plasma and the packed RBCs were frozen at −80°C for FA analysis. Another 500 μL was allocated for blood cell differentials, while the remaining 8.5 ml of the EDTA-collected blood was centrifuged at 930×g for 10 min at 4°C and the plasma aliquoted and frozen at −80°C for subsequent analysis. Based on the volume of plasma collected, the same volume of PBS was added back to the centrifuged cells, and white blood cells were obtained by density gradient centrifugation with Lymphoprep (StemCell Technologies, Vancouver, BC) as previously described (29). Complete blood counts were obtained with fresh whole blood collected in EDTA using a Coulter Ac•T dif2™ Hematology Analyzer (Beckman-Coulter Corp., Miami, FL). AST, ALT, Na+, K+, Cl-, bicarbonate, urea, estimated glomerular filtration rate (eGFR), creatinine, bilirubin, alkaline phosphatase and blood glucose were measured from blood collected in the SST gold tube at a commercial clinical lab (LifeLabs, Vancouver, BC).

### Inflammatory biomarkers measurements

The primary endpoint of this study was a reduction of plasma CRP levels. The secondary endpoints were changes in inflammatory cytokines in plasma and improvement of blood cell composition. Analyses of inflammatory markers were performed on stored, frozen plasma after the last participant completed their enrollment in the study. CRP was measured using a high sensitivity ELISA kit (R&D Systems, cat # DCRP00, Minneapolis, MN). The levels of fibrinogen, insulin, transforming growth factor-β (TGF-β), Leukotriene B_4_ (LTB_4_) and PGE_2_ in plasma were determined by ELISAs. These assays were performed using fibrinogen (cat # ab208036, Abcam), insulin (cat # DY8056, R&D Systems, Minneapolis, MN), LTB4 (cat # SKGE006B, R&D Systems, Minneapolis, MN) and PGE_2_ (cat # 514010, Cayman Chemical Company, Ann Arbor, MI) kits, according to the manufacturers' instructions. TGF-β (cat # 88-8350-88, Thermofisher Scientific, Ottawa, ON, Canada) levels were determined using a slight modification of the instructions. Specifically, to acid activate the latent TGF-β, 100 μL of each plasma sample was diluted with 25 μL of 1 N HCl for 10 min, followed by neutralization with fresh 1.2 N NaOH in 0.5 M HEPES. Standards were freshly prepared to reduce inter-assay variability.

Carcinoembryonic antigen (CEA) levels in the plasma were quantified using a Milliplex MAP Human Circulating Cancer Biomarker Panel from EMD Millipore (cat # hccbp1mag-58k, St. Louis, MO, USA). We also analyzed the plasma for the presence of 35 cytokines/chemokines using a 35-plex luminex assay (cat # LHC6005M, Thermofisher Scientific, Ottawa, ON, Canada). Because of the low levels of IL-6, IL-1β and TNF-α detected with this luminex assay, these cytokines were also quantified using a Mesoscale Discovery (MSD) V-Plex kit (cat # K151A9H-2, Mesoscale Discovery, Gaithersburg, MD), as previously described (30).

### Immunophenotyping

Peripheral blood mononuclear cells (PBMCs) were stained with antibodies against cell surface markers for 30 min at 23°C, washed twice with PFN, and resuspended with PFN for flow cytometry. For regulatory T cells (Tregs), cells stained with cell surface markers were fixed and permeabilized using the FOXP3 Staining Buffer Set (eBioscience, San Diego, CA). The cells were then stained with the FOXP3 antibody overnight at 4°C, washed once and resuspended with PFN and analyzed by flow cytometry. All flow cytometric analyses were performed using a BD LSR Fortessa flow cytometer (BD Biosciences). Data analysis was performed using FlowJo Software V10.3 (FlowJo, Ashland, OR). The antibodies used were CD3-FITC (clone SK7) from StemCell Technologies, Vancouver, BC, CD45-V500 (clone Hl30), CD4-PE Cy7 (clone SK3), CD127-A647 (clone HIL-7R-M21), CD25-BB515 (clone 2A3) all from BD Biosciences, Mississauga, ON, and CD14-PE (clone 61D3), CD56-APC (clone CMSSB), CD8-PercP Efluor780 (clone Y1/B2A), FOXP3-PE (clone 236a/E7) from Thermofisher Scientific (Ottawa, ON, Canada).

### Measurement of FA levels

The FA profile of the omega 3 supplement, the plasma and the packed RBC membrane samples was determined using gas chromatography with flame ionization detection (GC-FID) by Roger Dyer and Janette King at the BC Children's Hospital Research Institute, Vancouver, BC, as previously described (16).

### Statistical methods

The primary endpoint of this study was reduction of CRP at the end of 6 months of treatment with omega 3. The secondary endpoints were changes in the cytokines IL-6, IL-1β, TNFα, and TGFβ and the eicosanoid, PGE_2_, in plasma and changes in the plasma and RBC membrane concentration of EPA and DHA. Based on the results in the canakinumab study (12) where a reduction of 26–41% in CRP was observed, we estimated a sample size of 21 participants in each arm was needed to observe a 26% reduction in CRP in the treated group at 6 months with 80% power and a 2-sided type I error rate of 5%. The information from the second 6 months in the cross-over design would increase the power of the study, with each participant serving as his/her own control. The 6 months no treatment follow-up study in the intervention group would also inform the duration of the anti-inflammatory effect of omega 3 FAs. We enrolled 49 participants in our study to achieve the required number of participants since our rolling recruitment was interrupted by the COVID-19 pandemic, which led to a higher dropout rate than expected.

The characteristics of the volunteers in the control and the treatment group were compared using Fisher's exact test for categorical variables, and a *t*-test for continuous variables. To identify changes on a specific end point measure over time, we performed a mixed effect analysis with a Dunnet posthoc analysis, comparing values at a specific time point to baseline values in the treatment and the control groups. To compare the FA levels before and after supplementation in plasma and RBC membranes of the participants and the differences in the increases in EPA and DHA relative to baseline, we performed two-tailed paired *t*-tests. Pearson correlation analysis was performed to assess relationships between specific FAs and specific characteristics of the volunteers and other biomarkers. *P*-values lower than 0.05 were considered to be statistically significant. All statistical analyses were performed using GraphPad Prism 9.2.0.

## Results

### Fatty acid profile of the omega 3 capsules, the study cohort and their compliance

The FA profile of the omega 3 capsules was assessed, and the results confirmed the levels of EPA and DHA reported by the manufacturer (Supplementary Table 1). A total of 49 participants were recruited. Five participants in the control arm and 4 in the omega-3 arm dropped out due to reasons indicated in Figure 1. The dropout rate (20%) was higher than expected (~10%) due to COVID-19-related issues. The data from these participants were not included in the statistical analysis. Compliance with the intervention, as measured by capsule count, was high, averaging ~88.5%. The characteristics of the participants are shown in Supplementary Table 2.

### Omega 3 significantly increases plasma and RBC membrane levels of EPA and DHA

**Figure 2:**
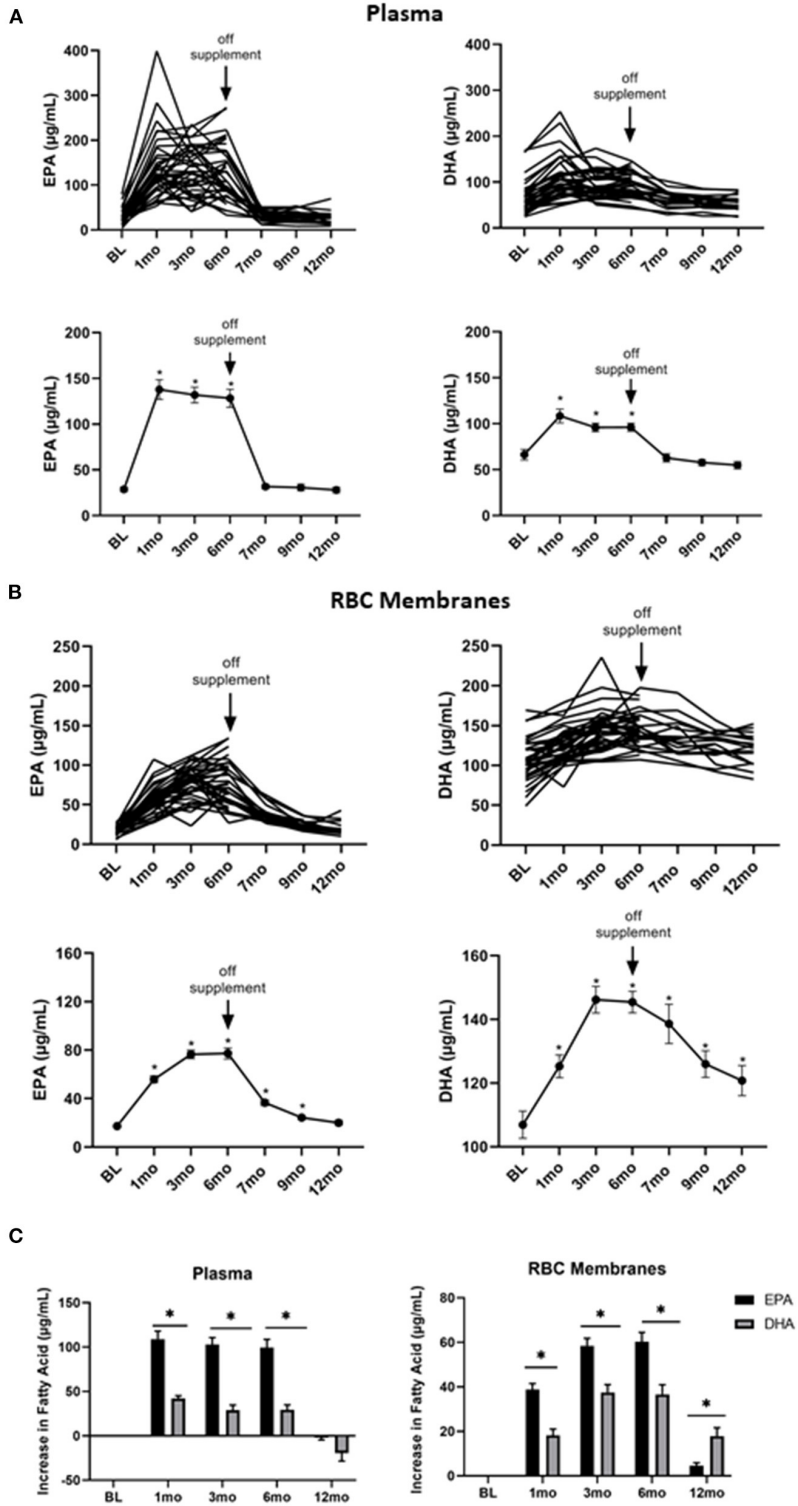
Omega 3 supplementation significantly increases plasma and RBC membrane levels of EPA and DHA, as determined by GC-FID. **(A)** Upper panel: Individual plasma EPA and DHA levels over time from participants taking omega 3 supplements. BL = baseline values. Omega 3 supplements were taken for the first 6 months. Lower panel: Average plasma EPA and DHA levels over time from participants taking omega 3 supplements. **(B)** Upper panel: Individual RBC membrane EPA and DHA levels over time from participants taking omega 3 supplements. BL = baseline values. Omega 3 supplements were taken for the first 6 months. Lower panel: Average RBC membrane EPA and DHA levels over time from participants taking omega 3 supplements. **(C)** Changes in EPA and DHA in plasma and RBC membranes over time relative to baseline values. All average values shown are the mean ± SEM. *denotes significant (*P* < 0.05) difference when compared to baseline values.

To determine the kinetics of uptake and clearance of EPA and DHA into plasma and cell membranes, plasma and RBC membrane levels of FAs were measured at baseline, 1, 3, 6, 7, 9, and 12 months. As shown in Figure 2A, both the individual and average plasma levels of EPA (left panel) and DHA (right panel) increased with treatment. They reached plateau levels within the first month and then quickly declined 1 month after stopping the supplements at the end of 6-months. However, EPA and DHA incorporation into RBC membranes, which has been shown to correspond to cell membrane levels in other cell types (31), was far slower, only plateauing 3 months after starting omega 3 and requiring 6 months (EPA) or longer (DHA) to return to baseline levels after stopping omega 3 (Figure 2B). Of note, the omega 3-induced increase in EPA from its baseline level was significantly (*P* < 0.0001) greater than that for DHA in both plasma and RBC membranes (Figure 2C), even though the peak DHA concentration was higher than EPA in RBC membranes (Figure 2B).

### Omega 3 significantly increases plasma and cell membrane levels of EPA + DHA/AA and the EPA levels achieved inversely correlate with BMI

**Figure 3:**
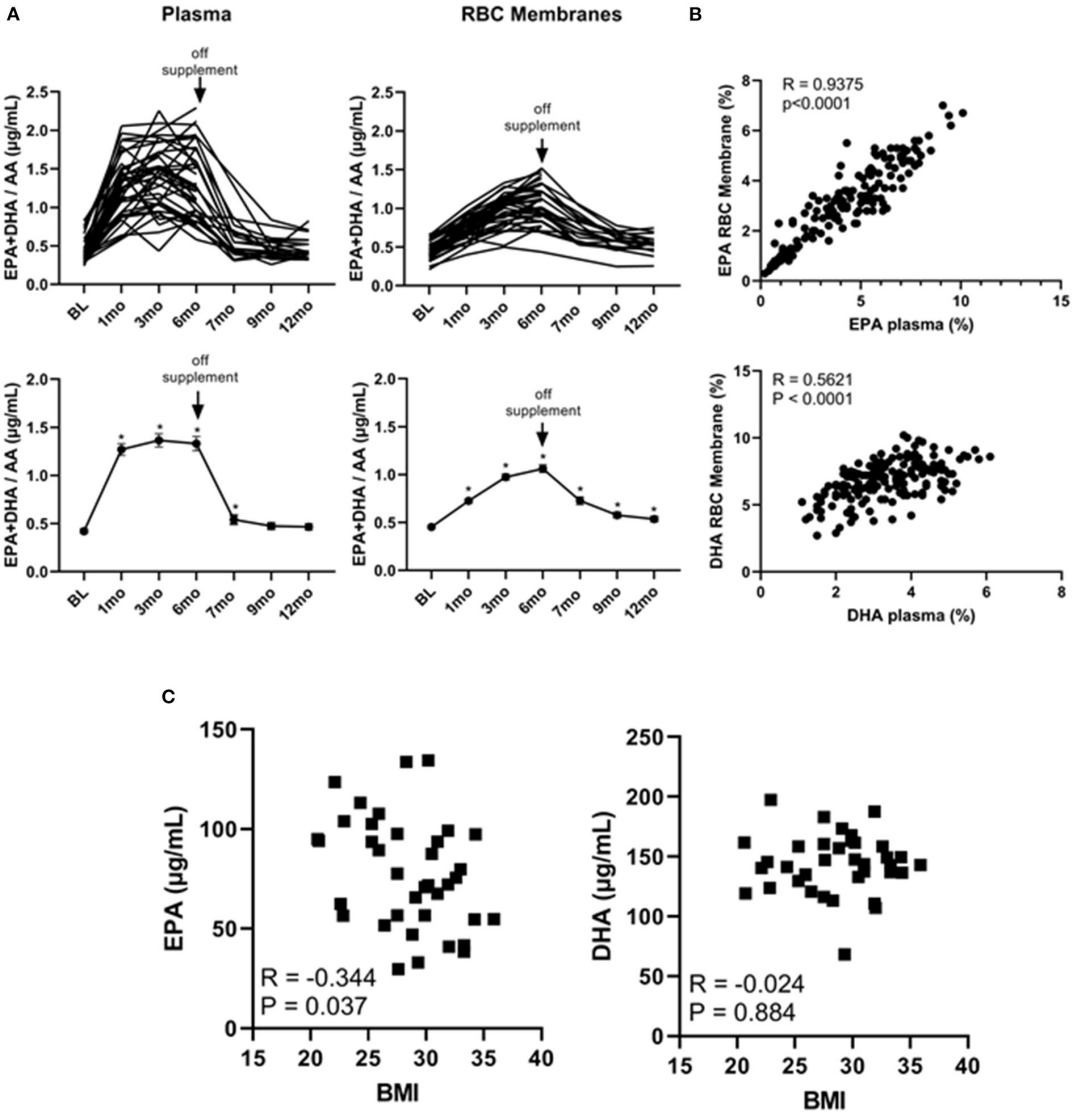
Omega 3 supplements significantly increase plasma and RBC membrane levels of EPA + DHA/AA and the RBC membrane EPA levels inversely correlate with initial BMI. **(A)** Upper panel: Individual plasma and RBC membrane EPA + DHA/AA levels over time from participants taking omega 3 supplements. Lower panel: Average plasma and RBC membrane EPA + DHA/AA levels over time from participants taking omega 3 supplements. All average values shown are the mean ± SEM. **(B)** Upper panel: Correlation between EPA levels (as a percent of total FAs) in RBC membranes and in plasma of participants during the omega 3 supplementation period. Lower panel: Correlation between DHA levels (as percent of total FAs) in RBC membranes and in plasma. **(C)** Correlation between the concentration of EPA or DHA (in μg/mL) in RBC membranes of participants throughout omega 3 supplements and their initial BMI. *Denotes significant (*P* < 0.05) difference when compared to baseline values.

It has been shown that the EPA + DHA/AA ratio within cell membranes plays a pivotal role in the production of immune modulators such as PGE_2_ (32, 33). We therefore plotted the individual and average EPA + DHA/AA ratios in both plasma and RBC membranes. As shown in Figure 3A, this ratio plateaued within 1 month in plasma but was still rising at the 6-month time point within RBC membranes. As well, after stopping the supplements at the 6-month time point, the EPA + DHA/AA ratio rapidly returned to baseline values in plasma but did not return to baseline values in RBC membranes even after cessation of the supplements for 6 months (at the 12-month time point). Because the DHA levels were still above baseline levels 6 months after stopping the omega 3 supplements, we could not use any values obtained after going off omega 3 for 6 months as control values. As a result, there were only 19 people in the control group as opposed to 39 in the active treatment group for the primary end-point analysis.

Importantly, as can be seen from the individual kinetic profiles in Figures 1, 2A, there was a large person-to-person variation in the levels of EPA and DHA in both plasma and RBC membranes. To gain some insight into the reasons for this large variation, we first compared individual plasma and RBC membrane levels. As shown in Figure 3B, we observed a significant (*P* < 0.0001) correlation between plasma and RBC membrane levels for EPA (*R* = 0.9375, *P* < 0.0001) as well as for DHA (*R* = 0.5621, *P* < 0.0001) during omega-3 treatment. The stronger correlation observed for EPA than DHA may simply reflect the higher concentration of EPA in the omega 3 and the lower baseline concentration of EPA in plasma and RBC membranes. We next looked for any correlations with sex, age and current vs. former smoker status but the only variable we found that could explain, to some extent, the large variation in EPA and DHA levels was BMI. Specifically, we found a significant inverse relationship between RBC membrane, but not plasma, EPA levels 6 months after taking omega 3 and BMI (measured at baseline), suggesting that incorporation of EPA (*R* = −0.343, *P* = 0.037), but, interestingly, not DHA (*R* = −0.025, *P* = 0.88), into cell membranes was reduced with increasing BMI (Figure 3C).

Of interest, our FA analysis revealed that after 6 months on omega 3 supplements, there was not only a significant increase in plasma and RBC membranes of EPA and DHA, but in the long chain omega 3 FA, Dpan-3 (C22:5n3) as well (Supplementary Table 3). This FA, also called docosapentanoic acid (DPA), has recently been demonstrated to have anti-inflammatory properties (34, 35). The observed increase in DPA could be due to its presence in the omega 3 capsules (~5% of total FA, Supplementary Table 1) and/or *via* enzymatic conversion from EPA (36). In addition, there was a significant reduction not only in plasma and cell membrane levels of AA but in other long-chain omega 6 FAs as well, such as Dpan-6 (C22:5n6), adrenic acid (C22:4n6) and homo-γ-linolenic acid (C20:3n6) (Supplementary Table 3).

### Omega 3 significantly reduces CRP levels

**Figure 4:**
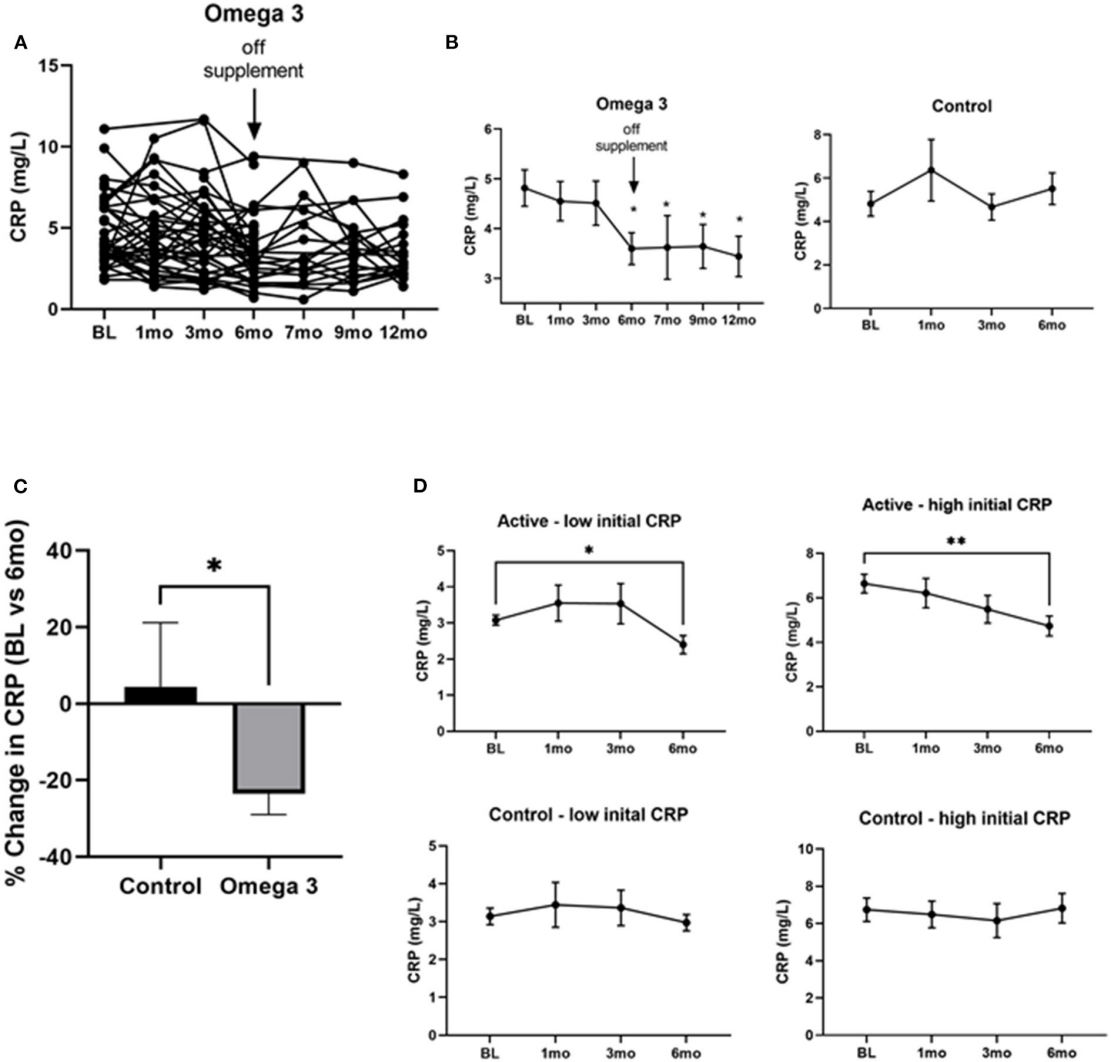
Omega 3 supplements significantly reduce CRP levels in participants with high initial CRP levels ≥4 mg/L. **(A)** Individual CRP levels of the participants on omega 3 for 6 months, as determined by high sensitivity ELISAs. **(B)** Average plasma CRP levels over time in the omega 3 treated group vs. the untreated group. All average values shown are the mean ± SEM of duplicate determinations. **(C)** Percent average change in CRP levels in the control and omega 3 group after 6 months, relative to their BL levels. **(D)** Changes in CRP levels in participants taking (active) and not taking omega 3 supplements (control) with low vs. high initial CRP levels. *Denotes significant (*P* < 0.05) difference when compared to baseline values. **Denotes significant (*P* < 0.01) difference when compared to baseline values.

To determine the effect of omega 3 supplements on inflammatory markers we first assessed CRP levels. As can be seen in Figure 4A, there was a large person-to-person variation in CRP levels. Despite this large variation, however, a statistically significant (*P* < 0.0004) decrease in CRP levels was observed 6 months after treatment with omega 3, when compared to their pre-treatment levels; the effect size for the CRP values at the end of the intervention relative to baseline was medium (Cohen's *d* = 0.56). This decrease was maintained for 6 months after stopping omega 3 (Figure 4B). A similar decrease was not observed in the untreated group (Figures 4B,C). The person-to-person variation in CRP levels after 6 months of omega 3 did not correlate with either the plasma or RBC membrane EPA, DHA or EPA + DHA levels. To determine if the effect of omega 3 on CRP was related to the initial level of CRP prior to treatment, we compared the effect of omega 3 in those who had initial values above or below the median CRP of 4.0 mg/L. We performed a similar analysis with the control group (median cut off of 3.9 mg/L). We found that the group that had high initial CRPs had a greater (*P* < 0.0029) reduction (~2.0 mg/L) in CRP levels 6 months after being on omega 3 than the participants who had low initial CRP levels (~0.6 mg/L) (Figure 4D). No significant changes were observed in the control group over time, regardless of their initial CRP levels (Figure 4D).

### Omega 3 significantly reduces PGE2 levels

**Figure 5:**
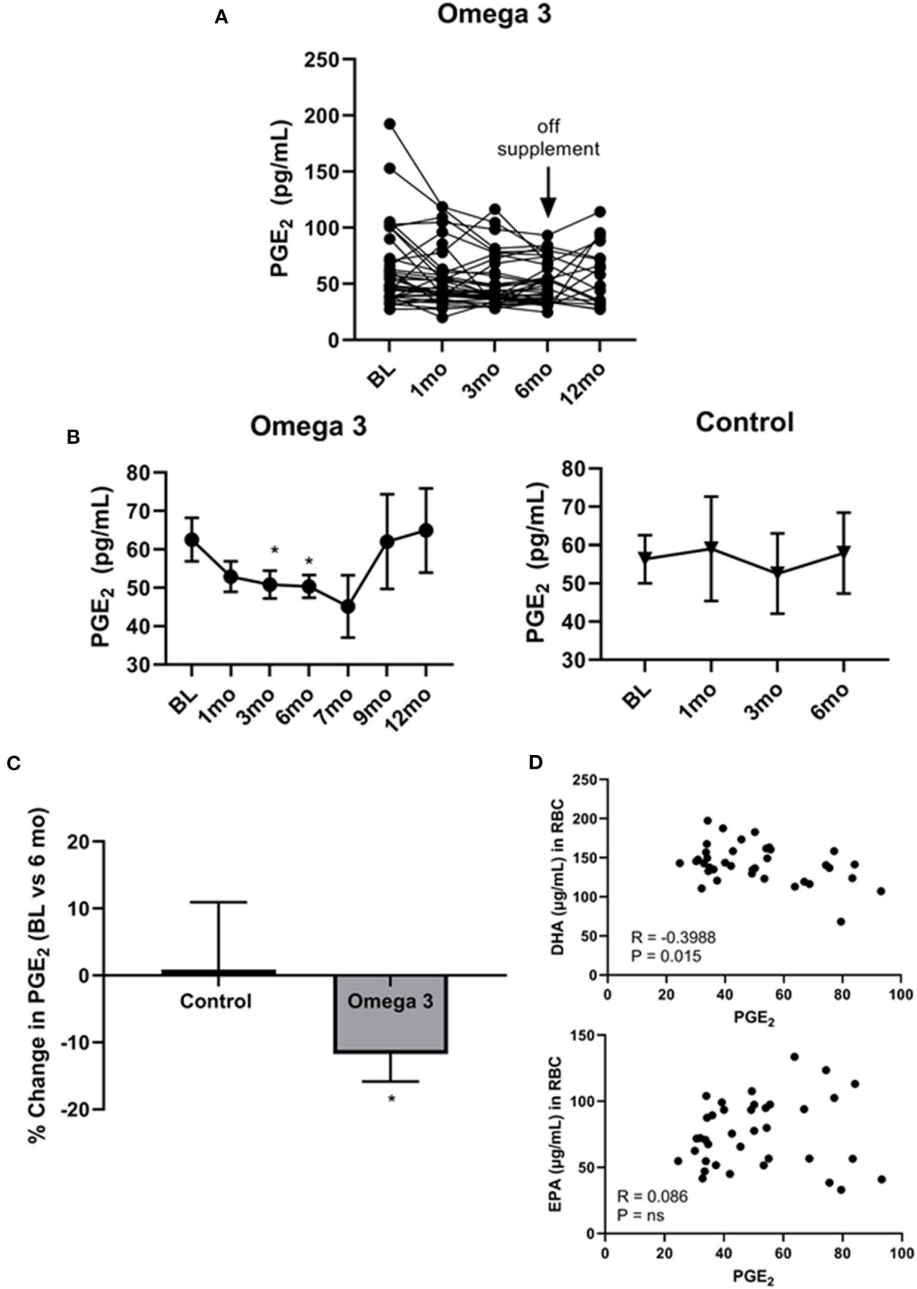
Omega 3 supplements significantly lower PGE_2_ levels. **(A)** Individual PGE_2_ levels of the participants on omega 3 supplements for 6 months as determined by PGE_2_ competitive ELISAs. **(B)** Average plasma PGE_2_ levels over time from participants taking omega 3 supplements for 6 months (left panel) and from the control group (right panel) over 6 months. All average values shown are the mean ± SEM. **(C)** Percent change in PGE_2_ levels after 6 months on omega 3 supplements, relative to baseline levels. **(D)** Correlation between EPA and DHA (μg/mL) levels in RBC membranes and PGE_2_ levels of participants consuming omega 3 supplements for 6 months. *Denotes significant (*P* < 0.05) difference when compared to baseline values.

Since cell membrane-incorporated EPA has been shown to compete with AA for COX2, generating the anti-inflammatory PGE_3_ rather than the pro-inflammatory PGE_2_ (32), we examined the effect of omega 3 on plasma PGE_2_ levels. As shown in Figure 5A, there was, once again, a large person-to-person variation in PGE_2_ levels and a decrease in average plasma PGE_2_ levels at 1 (*P* < 0.043), 3 (*P* < 0.014), and 6 months (*P* < 0.019) in the participants taking omega 3, relative to their baseline values (Figure 5B, left panel, C). No significant change was observed, however, in the control group (Figure 5B, right panel, C). Since the currently available PGE_2_ ELISAs also detect PGE_3_ (37), it is possible the observed effect of omega 3 on reducing PGE_2_ levels may be an underestimate. Worthy of note, PGE_2_ levels returned to baseline levels 3 months after stopping the supplements (at the 9-month time point). Correlation studies revealed a significant inverse relationship between PGE_2_ levels in those taking omega 3 for 6 months and their DHA levels (*R* = −0.3988, *P* = 0.0145), but not their EPA levels (Figure 5D). We also found that a decrease in AA was significantly associated with a decrease in PGE_2_ levels (*R* = 0.3330, *P* = 0.0440).

### Omega 3 does not significantly affect the levels of other tested cytokines/chemokines, LTB_4_, fasting blood glucose or insulin levels

We evaluated the effect of omega 3 on the plasma levels of IL-6, IL-1β, TNF-α, TGF-β, and LTB_4_ and found no significant effects (Supplementary Table 4, Figure 1). Similarly, there were no significant changes in the plasma levels of 35 cytokines/chemokines tested using a luminex assay (Supplementary Table 4). As well, there were no significant effects over time of omega 3 on fibrinogen or CEA levels (Supplementary Table 5), which were measured because these markers were found previously to be higher in people who smoke (30). There was also no significant change in either fasting blood glucose or insulin levels (Supplementary Table 5).

### Omega 3 significantly increases lymphocytes and decreases granulocytes

**Figure 6:**
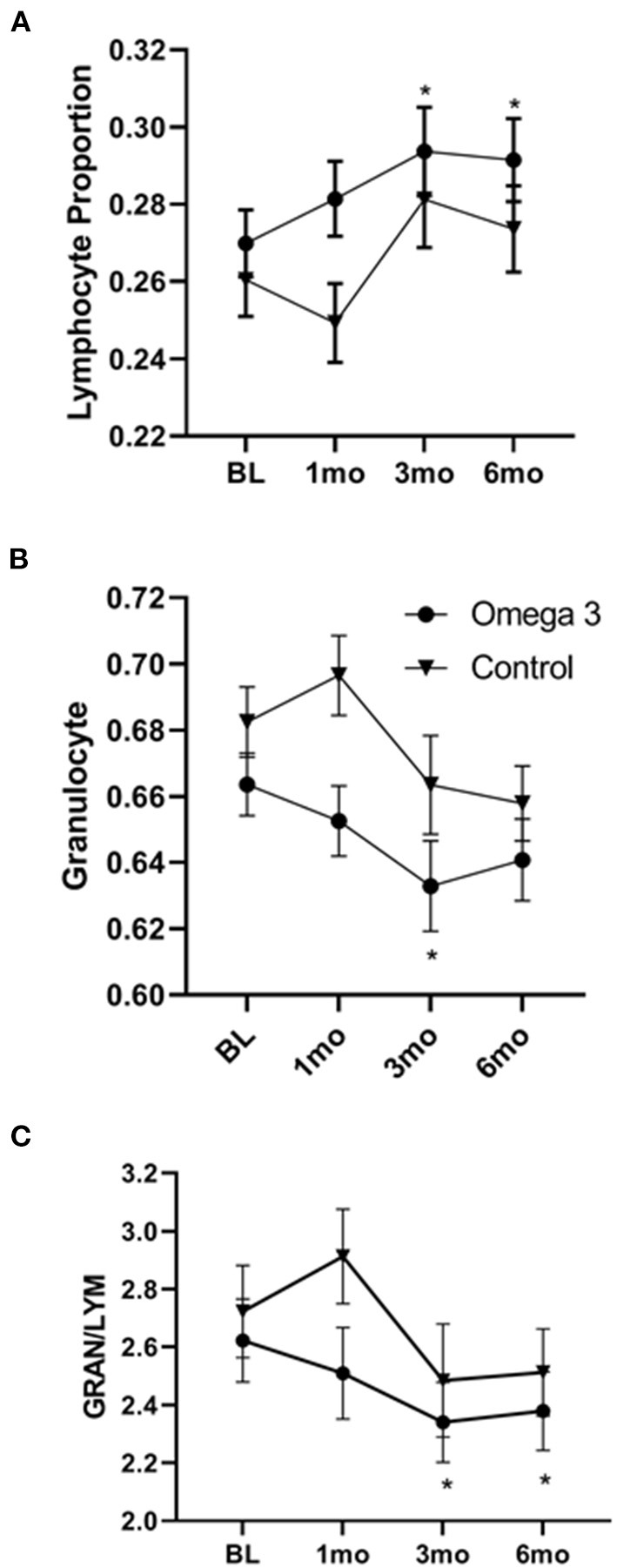
Omega 3 supplements significantly increase lymphocytes and decrease granulocytes, as determined by a hematology analyzer. **(A)** Average lymphocyte numbers, expressed as the proportion of total white blood cells, of participants taking (▾, *n* = 39) and not taking (•, *n* = 19) omega 3 supplements at baseline, 1, 3, and 6 months. **(B)** Average granulocyte numbers, expressed as the proportion of total white blood cells, of participants taking (▾, *n* = 39) and not taking (•, *n* = 19) omega 3 supplements at baseline, 1, 3, and 6 months. **(C)** Average granulocyte/lymphocyte ratio of participants taking (▾, *n* = 39) and not taking (•, *n* = 19) omega 3 supplements at baseline, 1, 3, and 6 months. *Denotes significant (*P* < 0.05) difference when compared to baseline values.

A significant increase in both the proportion and absolute numbers of lymphocytes was also observed 3 and 6 months after taking omega 3 (Figure 6A). There was also a reduction in the proportion of granulocytes, which reached significance 3 months after starting omega 3 (Figure 6B, Supplementary Table 6). As shown in Figure 6C, this resulted in a lower granulocyte/lymphocyte ratio in participants taking omega 3 supplements. We also observed transient decreases in regulatory T cells (Tregs), 1 month after starting omega 3 and in both total CD56 and CD56 dim natural killer cells, 1 and 3 months after beginning omega 3. However, we did not observe significant differences in these cell populations between the omega 3 treated and untreated groups after 6 months (Supplementary Table 7).

### Safety of omega 3 supplements

There was no significant change in the plasma levels of AST, ALT, Na+, K+, Cl–, bicarbonate, urea, creatinine, bilirubin and alkaline phosphatase during the 6 months of treatment with omega 3. A minor decrease in eGFR was observed 6 months after beginning omega 3. However, the levels remained within the normal range (Supplementary Table 8). The most commonly reported adverse events by the participants taking omega 3 were minor abdominal discomfort/constipation (Supplementary Table 9). Approximately 54% of participants taking omega 3 reported grade 1 flatulence/loose stool during follow up assessments, which was transient for about half the participants (28% resolved after 1–2 assessments) but was more chronic or recurring in others.

## Discussion

In this randomized cross-over clinical trial with participants in a lung cancer screening study with CRP levels >2 mg/L, omega 3 significantly lowered CRP levels (by 23%) after 6 months of supplementation compared to their pre-treatment levels. This reduction in CRP is close to that achieved by Canakinumab in patients with cardiovascular disease, who also had a significantly lowered incidence of lung cancer in a secondary analysis (12). With the dose of EPA and DHA given to the participants, we observed that EPA and DHA levels rose and plateaued within 1 and 3 months in plasma and RBC membranes, respectively, after starting omega 3. The peak levels of EPA and DHA achieved in the plasma and RBC membranes of our participants were comparable to those of earlier studies in which similar amounts of EPA + DHA were administered, both orally and intravenously (38–40). While the plasma levels of EPA and DHA dropped rapidly after discontinuation of omega 3, the RBC membrane levels of EPA and, especially, DHA, remained significantly higher 6 months after going off omega 3, suggesting a potentially long-lasting impact of omega 3 supplementation on RBC membrane DHA levels. Our finding that CRP levels did not go back up to pre-treatment levels 6 months after stopping the supplements (at month 12) would suggest that DHA, which also did not return to baseline levels at 12 months, may be more important than EPA at lowering CRP levels. An examination of the effects of omega 3 supplementation on participants with low or high initial CRP levels revealed that omega 3 had the most effect in participants who had higher initial CRP values ≥4 mg/L. Interestingly, in the Canakinumab trial, participants who had higher levels of CRP were the ones who appeared to benefit from Canakinumab treatment in reducing lung cancer incidence and mortality (12). It is thus possible that omega 3 supplements may be more impactful to those with a higher burden of CI.

We also found that EPA levels in RBC membranes were inversely correlated with the initial BMIs of the participants, suggesting that BMI may influence the efficacy of EPA supplementation. This is consistent with a previous study showing an inverse relationship between the accumulation of omega 3 FAs and body weight (41). It is thus reasonable to consider personalized doses based on body weight/BMI or a stratification factor in future randomized controlled trials. Looking at changes in FAs other than EPA and DHA, we also found that omega 3 supplementation significantly increased DPA levels in both plasma and RBC membranes. DPA is the third most prevalent omega 3 FA and has only recently garnered attention because of its presence both in the seal and whale meat consumed by the Inuits and in human breast milk (36, 42). Similar to EPA and DHA, DPA is thought to exhibit anti-inflammatory properties while at the same time serving as a potential storage depot for EPA and DHA (42). While DPA was not listed on the label of the Webber omega 3 supplement bottles, we identified 5% of the total FAs as DPA (Supplementary Table 1), which could explain the increase in DPA levels we observed with our active participants.

In the current study, we also found omega 3 supplements led to a reduction in PGE_2_, becoming significant within 1 month and continuing to be significantly lower than baseline levels at 3 and 6 months of supplementation. This decrease in PGE_2_ is expected since EPA and DHA compete with AA for incorporation into cell membranes. In addition, once released intracellularly by phospholipase A_2_, EPA and AA compete as substrates for COX2. AA is converted by COX2 to form the pro-inflammatory prostaglandin, PGE_2_ whereas EPA is converted to the anti-inflammatory prostaglandin, PGE_3_ (43, 44). DHA, on the other hand, may be converted to D series resolvins and neuroprotectins, which are anti-inflammatory and promote healing (45). It is important to note, however, that the effect of omega 3 on plasma PGE_2_ levels in the literature is mixed (39, 46–48). It is possible that the reduction in PGE_2_ we observed can be attributed not only to the dose of omega 3 we employed, which was higher than typically used in the literature, but also to the relatively long duration of our study. While it is tempting to speculate that DHA may play a more prominent role in reducing CRP while EPA may play a bigger role in regulating PGE_2_ levels, our finding that DHA (but not EPA) levels in RBC membranes inversely correlated with PGE_2_ levels may suggest that DHA may be the primary regulator of both inflammatory cytokines and PGE_2_ levels. The possibility that DHA is the primary regulator of inflammatory cytokines is in keeping with a recent report by So et al. (49) comparing the effects of giving either 3 g/day of EPA or DHA to 50–75 year old volunteers with chronic inflammation. They found that DHA had broader anti-inflammatory properties than EPA upon testing *ex vivo* monocyte inflammatory responses. Future studies focusing on the efficacy of EPA vs. DHA vs. the two together (to explore synergism) on the inflammatory status of heavy smokers at high risk of lung cancer are thus warranted.

Based on both our previous study showing that 30 people who smoked heavily (16 men and 14 women) had significantly higher plasma CRP, fibrinogen, IL-6 and CEA levels than 36 controls who had never smoked, and other studies (50), we were surprised that we did not see any significant effects of omega 3 on the levels IL-6 and IL-1β, as well as many other pro-inflammatory cytokines. However, this lack of effect has also been reported by several other groups (51, 52). Specifically, Blok et al. (51), who gave participants similar doses of EPA + DHA (i.e., 2.43 g EPA + 0.49 g DHA/day) to that used in our study for 1 year found no significant differences in circulating TNF-α and IL-1β level. Other studies in which subjects with metabolic syndrome or persistent atrial fibrillation were given up to 4 g/day of EPA + DHA (i.e., 1.8–2.1 g EPA + 1.5 g DHA/day) for as long as 24 weeks also did not find significant changes in circulating cytokines/chemokines (53, 54).

Since LTB_4_ is a pro-inflammatory lipid mediator that is also derived from AA, we quantified plasma levels of LTB_4_ in participants before and after omega 3 supplementation and found no significant changes. This lack of correlation between PGE_2_ and LTB_4_ levels is consistent with previously reported observations in prostate cancer tissues (55). Considering that omega 3 supplementation also did not impact inflammatory cytokines other than CRP, it is possible that the effects of EPA and DHA are specific to CRP and PGE_2_ levels.

In the current study, omega 3 supplementation was found to decrease the proportion of granulocytes and increase lymphocytes. This omega 3 FA-induced reduction in the granulocyte/lymphocyte ratio is of interest since our previous studies with people who smoke showed they had increased granulocytes compared to people who did not smoke (56) and there is evidence in the literature that a high granulocyte/lymphocyte ratio correlates with inflammation (57) and cancer risk (58). This suggests that omega 3 might be effective in preventing lung cancer in heavy smokers.

Our study is unique compared to previous studies, given that the concentrations of EPA Our study is unique compared to previous studies, given that the concentrations of EPA and DHA used were at the high end of the spectrum and the study was longer than most studies. We also quantified the levels of FAs in both plasma and RBC membranes over time and characterized the effects of omega 3 over time on multiple markers of inflammatory status and immune cell profile in these smokers. This is important since many studies evaluating the efficacy of omega 3 do not report the level of EPA or DHA achieved in plasma and RBC membranes. Our study also has several limitations. First, we did not include an adequate washout period, given that DHA was still, unexpectedly, elevated in RBC membranes 6 months after the participants stopped their supplements. This resulted in fewer participants in the untreated arm for comparison. Second, we did not find a significant correlation between changes in CRP levels and the EPA/DHA levels in plasma and RBC membranes, even though omega 3 supplementation significantly lowered CRP and increased EPA/DHA levels. This lack of a relationship might be attributable to the dramatic person-to-person variation that we observed in both CRP and EPA/DHA levels. Specifically, it is possible that individuals respond very differently in terms of CRP production in response to the same levels of EPA and DHA in their plasma or RBC membranes. This variation would obscure the relationship between EPA/DHA and CRP levels.

## Conclusion

In conclusion, our results suggest that omega 3 supplements taken at 3.6 g/days lower the inflammatory markers CRP and PGE_2_ but not other pro-inflammatory markers. Future studies evaluating the efficacy of EPA + DHA vs. DHA alone and EPA alone are needed to understand if one of these FAs is more potent than the other in modulating inflammatory responses or if they work synergistically. Our findings support a future longer-term randomized control trial to look at the effects of omega 3 in reducing the incidence of lung cancer in people who are at high risk for lung cancer and have high levels (>2 mg/L) of the inflammatory marker, CRP.

## Data availability statement

The raw data supporting the conclusions of this article will be made available by the authors, without undue reservation.

## Ethics statement

The studies involving human participants were reviewed and approved by Clinical Research Ethics Board of the University of British Columbia and BC Cancer (H19-00221). The patients/participants provided their written informed consent to participate in this study.

## Author contributions

IE: conceptualization, formal analysis, investigation, writing-original draft, writing- review and editing, visualization, and supervision. MY and SK: formal analysis, investigation, writing-review and editing, and visualization. JW and HR: investigation. RD: investigation and writing-review and editing. SA-K**:** investigation and project administration. SL: conceptualization, writing-review and editing, resources, supervision, and funding acquisition. GK: conceptualization, writing-original draft, writing-review and editing, resources, supervision, and funding acquisition. All authors contributed to the article and approved the submitted version.

## Funding

This work was funded by Hecht Grant ID #4429 to GK and SL. The funder had no role in the design of the study, in the collection, analysis, interpretation of the data, in the writing process of the manuscript, and decision to submit the manuscript for publication.

## Conflict of interest

The authors declare that the research was conducted in the absence of any commercial or financial relationships that could be construed as a potential conflict of interest.

## Publisher's note

All claims expressed in this article are solely those of the authors and do not necessarily represent those of their affiliated organizations, or those of the publisher, the editors and the reviewers. Any product that may be evaluated in this article, or claim that may be made by its manufacturer, is not guaranteed or endorsed by the publisher.
