# Supplementary material for: Omega 3 supplementation reduces C-reactive protein, prostaglandin E2 and the granulocyte/lymphocyte ratio in heavy smokers: An open-label randomized crossover trial
Source: Front Nutr. 2022 Dec 1;9:1051418. doi: 10.3389/fnut.2022.1051418 (PMC9751896; doi:10.3389/fnut.2022.1051418)
Supplement: Supplementary file 1 [file Table_1.DOC]

**Supplementary Table 1**. **Fatty acid profile of the omega 3 capsules**

| Fatty Acid | % FA |
| --- | --- |
| C 12: 0 | 0.0 |
| C 14: 0 | 0.1 |
| C 14: 1 | 0.0 |
| C 16: 0/  Palmitic acid | 0.1 |
| C 16: 1n9 | 0.0 |
| C 16: 1n7 | 0.1 |
| C 18: 0/  Stearic acid | 1.4 |
| C 18: 1n9/  Oleic acid | 1.6 |
| C 18: 1n7 | 0.8 |
| C 18: 2n6/  Linoleic acid | 0.2 |
| C 18: 3n6/  γlinoleic acid | 0.0 |
| C 20: 0 | 1.1 |
| C 18: 3n3/  αlinolenic acid | 0.1 |
| C 20: 1n11 | 3.9 |
| C 20: 1n9 | 0.5 |
| C 18: 4n3 | 0.4 |
| C 20: 2n6/  Eicosadienoic acid | 0.3 |
| C 20: 3n9 | 0.1 |
| C20: 3n6/  Eicosatrienoic acid | 0.4 |
| C 22: 0 | 0.2 |
| C 20: 4n6/  Arachidonic acid C 20: 5n3 | 3.5  50.5 |
| C 24: 0 | 0.3 |
| C 22: 4n6/  Adrenic acid | 2.4 |
| C 24: 1 C 22: 5n6 C 22:5n3 | 0.8  0.2  5.0 |
| C 22: 6n3/  Docosahexaenoic acid | 25.8 |
