# Supplementary material for: Omega 3 supplementation reduces C-reactive protein, prostaglandin E2 and the granulocyte/lymphocyte ratio in heavy smokers: An open-label randomized crossover trial
Source: Front Nutr. 2022 Dec 1;9:1051418. doi: 10.3389/fnut.2022.1051418 (PMC9751896; doi:10.3389/fnut.2022.1051418)
Supplement: Supplementary file 2 [file Table_2.DOCX]

**Supplementary Table 2. Characteristics of participants.**

| **Characteristic** | **Omega 3-Treated Group (n = 20)** | **Untreated – Controls**  **(n = 19)** | **P-value** |
| --- | --- | --- | --- |
| Age (years) | 64.2 ± 1.5 | 64.2 ± 1.7 | 0.71 |
| Male % | 40.0% | 35.0% | >0.99 |
| BMI | 29.1 ± 0.9 | 29.6 ± 1.7 | 0.86 |
| Current smokers % | 25% | 40.0% | 0.50 |
| Pack years | 48.1 ± 4.9 | 42.8 ± 4.3 | 0.60 |
