# Supplementary material for: Omega 3 supplementation reduces C-reactive protein, prostaglandin E2 and the granulocyte/lymphocyte ratio in heavy smokers: An open-label randomized crossover trial
Source: Front Nutr. 2022 Dec 1;9:1051418. doi: 10.3389/fnut.2022.1051418 (PMC9751896; doi:10.3389/fnut.2022.1051418)
Supplement: Supplementary file 3 [file Table_3.DOC]

|  | RBC membranes (μg/mL) | | Plasma (μg/mL) | | |
| --- | --- | --- | --- | --- | --- |
| Fatty Acid | Baseline | 6 Months | | Baseline | 6 Months |
| C 12: 0 | 0.1 ± 0.1 | 0.1 ± 0.2 | | 0.0 ± 0.0 | 0.0 ± 0.0 |
| C 14: 0 | 5.1 ± 2.3 | 4.3 ± 1.7 * | | 24.2 ± 11.9 | 20.5 ± 10.7 * |
| C 14: 1 | 0.2 ± 0.1 | 0.2 ± 0.1 | | 2.4 ± 1.6 | 1.7 ± 1.2 * |
| C 16: 0/  Palmitic acid | 350.7 ± 45.1 | 344.8 ± 36.8 | | 651.6 ± 187.2 | 574.8 ± 132.6 * |
| C 16: 1n9 | 2.1 ± 1.3 | 1.5 ± 0.5 * | | 10.9 ± 5.1 | 7.9 ± 1.9 * |
| C 16: 1n7 | 11.8 ± 8.3 | 8.6 ± 4.9 * | | 67.0 ± 39.6 | 51.6 ± 31.6 * |
| C 18: 0/  Stearic acid | 253.8 ± 20.6 | 252.6 ± 17.3 | | 251.8 ± 59.1 | 238.1 ± 59.7 |
| C 18: 1n9/  Oleic acid | 279.6 ± 60.1 | 258.3 ± 30.5 | | 569.5 ± 191.4 | 447.3 ± 114.3 * |
| C 18: 1n7 | 23.3 ± 6.5 | 21.6 ± 4.0 | | 73.7 ± 21.9 | 61.4 ± 17.1 * |
| C 18: 2n6/  Linoleic acid | 216.0 ± 66.0 | 178.3 ± 44.4 * | | 670.0 ± 205.9 | 540.0 ± 112.9 * |
| C 18: 3n6/  γlinoleic acid | 2.3 ± 1.1 | 1.4 ± 0.6 * | | 11.6 ± 5.7 | 6.4 ± 3.2 * |
| C 20: 0 | 4.0 ± 1.2 | 4.3 ± 1.0 * | | 2.5 ± 1.3 | 2.5 ± 0.9 |
| C 18: 3n3/  αlinolenic acid | 5.0 ± 3.0 | 3.9 ± 1.5 * | | 20.8 ± 12.7 | 17.1 ± 7.7 |
| C 20: 1n11 | 6.7 ± 1.3 | 5.5 ± 0.8 * | | 6.2 ± 1.7 | 5.0 ± 1.3 * |
| C 20: 1n9 | 0.6 ± 0.3 | 0.6 ± 0.2 | | 0.8 ± 0.6 | 0.8 ± 0.6 |
| C 18: 4n3 | 0.2 ± 0.2 | 0.1 ± 0.1 * | | 0.6 ± 0.9 | 0.2 ± 0.6 * |
| C 20: 2n6/  Eicosadienoic acid | 4.4 ± 1.2 | 3.5 ± 0.8 * | | 5.7 ± 2.8 | 4.2 ± 1.4 * |
| C 20: 3n9 | 2.0 ± 0.7 | 1.3 ± 0.4 * | | 5.2 ± 2.4 | 2.6 ± 1.1 * |
| C20: 3n6/  Eicosatrienoic acid | 28.9 ± 5.3 | 21.0 ± 4.4 * | | 47.3 ± 15.6 | 31.7 ± 8.9 * |
| C 22: 0 | 10.2 ± 4.3 | 11.1 ± 4.0 | | 4.7 ± 3.5 | 4.0 ± 1.4 |
| C 20: 4n6/  Arachidonic acid | 270.3 ± 30.7 | 211.1 ± 28.2 * | | 215.9 ± 55.6 | 172.5 ± 37.3 * |
| C 20: 5n3/  Eicosapentaenoic acid 17.1 ± 4.7 77.3 ± 27.0 * 29.1 ± 15.3 126.6 ± 61.9 * | | | | | |
| C 24: 0 | 27.8 ± 12.1 | 32.8 ± 13.5 * | | 5.6 ± 3.2 | 5.2 ± 2.0 |
| C 22: 4n6/  Adrenic acid | 51.9 ± 8.2 | 27.7 ± 6.4 * | | 5.7 ± 1.9 | 2.8 ± 1.0 * |
| C 24: 1 | 33.6 ± 14.5 | 35.6 ± 14.2 | | 9.3 ± 6.8 | 7.1 ± 2.3 * |
| C 22: 5n6/  Docosapentaenoic-6 acid 7.7 ± 1.7 3.7 ± 1.5 * 3.3 ± 1.4 1.6 ± 0.6 * | | | | | |
| C 22: 5n3/  Docosapentaenoic-3 acid 44.7 ± 6.9 71.7 ± 10.6 * 19.8 ± 7.3 30.9 ± 11.6 * | | | | | |
| C 22: 6n3/  Docosahexaenoic acid | 106.0 ± 27.0 | 142.6 ± 24.5 * | | 67.5 ± 37.0 | 96.8 ± 26.9 * |

**Supplementary Table 3**. **Fatty acid profile in plasma and RBC membranes of participants (n = 39) at baseline and after taking omega 3 for 6 months.**

* denotes significant (P<0.05) difference when compared to baseline values.
