# Supplementary material for: Omega 3 supplementation reduces C-reactive protein, prostaglandin E2 and the granulocyte/lymphocyte ratio in heavy smokers: An open-label randomized crossover trial
Source: Front Nutr. 2022 Dec 1;9:1051418. doi: 10.3389/fnut.2022.1051418 (PMC9751896; doi:10.3389/fnut.2022.1051418)
Supplement: Supplementary file 4 [file Table_4.DOCX]

**Supplemental Table 4. The effect of omega 3 supplements on selected inflammatory markers (n = 39).**

**Active Control**

**Analyte (pg/mL) BL 1mo 3mo 6mo BL 1mo 3mo 6mo**

EGF 17.6 ± 1.6 19.4 ± 1.3 15.2 ± 0.9 18.0 ± 1.3 17.8 ± 1.5 17.8 ± 1.4 21.1 ± 3.9 16.3 ± 1.6

Eotaxin 33.9 ± 2.0 35.7 ± 2.1 34.9 ± 2.2 35.6 ± 1.6 30.3 ± 2.8 32.5 ± 3.2 34.2 ± 2.9 33.8 ± 2.7

FGF-Basic 6.5 ± 0.3 6.5 ± 0.3 6.4 ± 0.3 6.4 ± 0.3 6.5 ± 0.2 7.4 ± 0.8^*^ 8.1 ± 1.1 5.2 ± 0.2^*^
G-CSF 69.7 ± 4.9 76.6 ± 6.2 62.8 ± 4.4 70.3 ± 6.5 55.5 ± 6.0 57.8 ± 8.0 55.8 ± 9.0 62.2 ± 5.5

GM-CSF 8.9 ± 0.9 10.3 ± 1.0 7.9 ± 0.6 10.0 ± 1.2 5.1 ± 0.4 5.3 ± 0.5 5.5 ± 0.7 6.5 ± 0.6

HGF 100.3 ± 8.3 109.3 ± 13.4 90.9 ± 6.0 95.3 ± 6.6 78.2 ± 10.7 82.1 ± 10.4 75.7 ± 10.3 77.4 ± 4.0

IFN-α 19.0 ± 2.0 20.5 ± 2.7 16.6 ± 0.6 18.4 ± 1.3 18.1 ± 1.7 18.1 ± 1.5 18.3 ± 1.8 16.3 ± 0.9

IL-1β 0.1 ± 0.0 0.1 ± 0.0 0.1 ± 0.0 0.1 ± 0.0 0.1 ± 0.0 0.1 ± 0.0 0.1 ± 0.0 0.1 ± 0.0

IL-1RA 35.3 ± 3.9 37.0 ± 5.5 32.8 ± 3.2 33.1 ± 2.9 38.2 ± 8.6 32.5 ± 6.2 33.4 ± 7.0 30.4 ± 3.5

IL-2 13.3 ± 0.8 14.1 ± 1.1 12.2 ± 0.5 12.9 ± 0.6 11.8 ± 1.3 11.9 ± 1.2 11.5 ± 1.2 12.1 ± 0.6

IL-2R 101.0 ± 18.0 115.4 ± 21.6 106.5 ± 18.8 82.7 ± 7.1 88.7 ± 19.8 93.5 ± 19.3 87.5 ± 18.6 88.0 ± 13.7

IL-3 11.5 ± 1.2 12.5 ± 1.1 10.5 ± 0.9 10.9 ± 0.9 10.2 ± 0.5 9.6 ± 0.5 10.1 ± 0.7 9.4 ± 0.6

IL-6 1.2 ± 0.1 1.2 ± 0.1 1.2 ± 0.1 1.1 ± 0.1 1.2 ± 0.1 1.1 ± 0.1 1.0 ± 0.1 1.3 ± 0.1

IL-7 7.2 ± 0.8 7.7 ± 1.0 7.7 ± 1.0 6.5 ± 0.3 7.2 ± 1.6 7.5 ± 1.5 7.2 ± 1.4 6.3 ± 0.3

IL-8 5.5 ± 0.4 5.2 ± 0.3 4.8 ± 0.3 5.0 ± 0.3 4.9 ± 0.3 4.5 ± 0.3 4.8 ± 0.3 5.1 ± 0.3

IL-10 28.0 ± 3.8 28.3 ± 3.3 26.1 ± 3.9 30.0 ± 4.4 16.2 ± 0.7 16.1 ± 0.7 15.7 ± 0.8 19.4 ± 1.0^*^

IL-12 55.7 ± 4.3 59.2 ± 4.6 57.8 ± 4.4 56.7 ± 3.7 67.7 ± 6.1 71.7 ± 7.5 64.3 ± 5.1 53.5 ± 4.5^*^

IL-22 16.4 ± 1.2 17.9 ± 1.5 16.6 ± 1.8 18.1 ± 1.8 15.2 ± 2.8 16.1 ± 3.8 21.8 ± 6.8 13.6 ± 0.6

IP-10 15.9 ± 1.4 18.2 ± 1.7 16.9 ± 1.4 16.1 ± 1.5 15.5 ± 1.9 16.1 ± 2.2 12.7 ± 1.8 15.5 ± 2.2

MCP-1 197.5 ± 14.4 201.9 ± 14.4 197.5 ± 14.6 201.3 ± 12.7 189.2 ± 20.2 176.5 ± 17.0 187.9 ± 19.3 210.3 ± 19.9

RANTES 527.9 ± 27.3 544.6 ± 26.8 543.0 ± 26.6 552.8 ± 21.9 787.0 ± 81.8 780.1 ± 71.1 817.2 ± 112.5 572.2 ± 42.4^*^

TGF-β 4.8 ± 0.2 4.8 ± 0.2 4.3 ± 0.2 4.8 ± 0.3 5.1 ± 0.5 4.6 ± 0.3 4.6 ± 0.3 5.0 ± 0.4

TNF-α 1.3 ± 0.1 1.3 ± 0.1 1.3 ± 0.1 1.3 ± 0.1 1.2 ± 0.1 1.2 ± 0.1 \ 1.1 ± 0.0 1.4 ± 0.1

VEGF 12.2 ± 0.9 12.5 ± 1.1 11.2 ± 0.9 11.2 ± 0.9 11.4 ± 1.7 11.3 ± 1.6 12.4 ± 2.3 11.3 ± 1.2

* denotes a significant (P<0.05) difference when compared to baseline values.
