# Supplementary material for: Omega 3 supplementation reduces C-reactive protein, prostaglandin E2 and the granulocyte/lymphocyte ratio in heavy smokers: An open-label randomized crossover trial
Source: Front Nutr. 2022 Dec 1;9:1051418. doi: 10.3389/fnut.2022.1051418 (PMC9751896; doi:10.3389/fnut.2022.1051418)
Supplement: Supplementary file 5 [file Table_5.DOCX]

**Supplemental Table 5. The effect of omega 3 supplements on CEA, fibrinogen, insulin and blood glucose.**

**Active (n = 39) Placebo (n = 19)**

**BL 1mo 3mo 6mo BL 1mo 3mo 6mo**

CEA (pg/mL) 660.5 ± 91.6 713.5 ± 103.4 747.9 ± 108.6^*^ 734.3 ± 121.2 602.9 ± 116.1 685.8 ± 170.9 660.2 ± 187.5 814.9 ± 166.9^*^

Fibrinogen 5242 ± 226.9 4579 ± 217.6^*^ 4851 ± 164.9 4909.0 ± 179.7 4657.0 ± 165.1 4786.0 ± 180.6 4845.0 ± 180.6 4958.0 ± 209.1
(µg/mL)

Insulin (µg/L) 63.0 ± 5.0 69.6 ± 5.9 67.8 ± 6.2 68.1 ± 5.8 58.1 ± 9.6 56.8 ± 9.4 52.3 ± 7.8 66.5 ± 8.7

Blood glucose 5.4 ± 0.1 5.3 ± 0.1 5.2 ± 0.1 5.6 ± 0.1 5.2 ± 0.1 5.0 ± 0.2 5.1 ± 0.2 5.4 ± 0.2
(mmol/L)

* denotes a significant (P < 0.05) difference when compared to baseline values.
