# Supplementary material for: Omega 3 supplementation reduces C-reactive protein, prostaglandin E2 and the granulocyte/lymphocyte ratio in heavy smokers: An open-label randomized crossover trial
Source: Front Nutr. 2022 Dec 1;9:1051418. doi: 10.3389/fnut.2022.1051418 (PMC9751896; doi:10.3389/fnut.2022.1051418)
Supplement: Supplementary file 6 [file Table_6.DOCX]

**Supplementary Table 6. Complete Blood Count Results from Participants Taking Omega 3 Supplements and Controls.**

**Active (n = 39)**   **Control (n = 19)**

**BL 1mo 3mo 6 mo BL 1mo 3mo 6 mo**

WBC (x10^9^/L) 6.5 ± 0.2 6.5 ± 0.2 6.6 ± 0.3 6.7 ± 0.3 6.9 ± 0.4 7.0 ± 0.5 6.7 ± 0.3 6.4 ± 0.3

LYM^‡^ 0.3 ± 0.0 0.3 ± 0.0 0.3 ± 0.0^*^ 0.3 ± 0.0^*^ 0.3 ± 0.0 0.2 ± 0.0 0.3 ± 0.0 0.3 ± 0.0

MON^‡^  0.1 ± 0.0 0.1 ± 0.0 0.1 ± 0.0 0.1 ± 0.0 0.1 ± 0.0 0.1 ± 0.0 0.1 ± 0.0 0.1 ± 0.0^*^

GRAN^‡^ 0.7 ± 0.0 0.7 ± 0.0 0.6 ± 0.0^*^ 0.6 ± 0.0 0.7 ± 0.0 0.7 ± 0.0 0.7 ± 0.0 0.7 ± 0.0

LYM# (x10^9^/L) 1.7 ± 0.1 1.8 ± 0.1 1.9 ± 0.1^*^ 1.9 ± 0.1^*^ 1.7 ± 0.1 1.7 ± 0.1 1.8 ± 0.1 1.8 ± 0.1

MON# (x10^9^/L) 0.4 ± 0.0 0.4 ± 0.0 0.4 ± 0.0 0.4 ± 0.0 0.4 ± 0.0 0.4 ± 0.0 0.4 ± 0.0 0.4 ± 0.0

GRAN# (x10^9^/L) 4.3 ± 0.2 4.2 ± 0.2 4.2 ± 0.2 4.4 ± 0.2 4.6 ± 0.3 4.9 ± 0.4 4.5 ± 0.3 4.2 ± 0.2

RBC (x10^12^/L) 5.0 ± 0.1 4.8 ± 0.1 5.0 ± 0.1 5.0 ± 0.1 5.0 ± 0.1 4.9 ± 0.1 4.9 ± 0.2 4.9 ± 0.1

HGB (g/L) 154.7 ± 3.0 148.5 ± 2.6 151.2 ± 2.4 152.4 ± 2.8 154.4 ± 4.4 148.6 ± 3.7 145.8 ± 3.5 152.2 ± 3.6

HCT (L/L) 0.5 ± 0.0 0.4 ± 0.0 0.4 ± 0.0 0.5 ± 0.0 0.5 ± 0.0 0.4 ± 0.0 0.4 ± 0.0 0.4 ± 0.0

MCV (fL) 90.8 ± 0.9 90.9 ± 0.9 90.4 ± 0.9 90.9 ± 0.8 90.7 ± 1.3 90.8 ± 1.4 90.2 ± 1.1 90.7 ± 1.6

MCH (pg) 30.9 ± 0.3 31.1 ± 0.4 30.6 ± 0.3^*^ 30.8 ± 0.3 30.9 ± 0.5 30.4 ± 0.5 30.0 ± 0.6^*^ 31.0 ± 0.5

MCHC (g/L) 340.7 ± 1.3 341.6 ± 1.5 337.2 ± 1.7 338.6 ± 1.5 341.0 ± 2.3 335.1 ± 1.3^*^ 332.6 ± 2.8 341.6 ± 2.4

RDW (%) 13.3 ± 0.1 13.3 ± 0.1 13.1 ± 0.1 13.3 ± 0.1 13.3 ± 0.2 13.3 ± 0.2 13.1 ± 0.2 13.5 ± 0.2

PLT (x10^9^/L) 208.7 ± 9.9 212.7 ± 8.7 218.1 ± 9.2 220.3 ± 8.9 205.8 ± 14.8 225.9 ± 17.0 235.6 ± 18.9 217.7 ± 13.8

MPV (f/L) 8.6 ± 0.1 8.5 ± 0.1 8.5 ± 0.1 8.5 ± 0.2 8.4 ± 0.1 8.5 ± 0.2 8.6 ± 0.2 8.3 ± 0.2

PCT (%) 0.2 ± 0.0 0.2 ± 0.0 0.2 ± 0.0 0.2 ± 0.0 0.2 ± 0.0 0.2 ± 0.0 0.2 ± 0.0^*^ 0.2 ± 0.0

PDW 16.0 ± 0.1 15.9 ± 0.1 15.9 ± 0.1 16.0 ± 0.1 16.1 ± 0.1 16.0 ± 0.1 15.9 ± 0.1 16.0 ± 0.1

* denote significant difference (P < 0.05) compared to baseline values. Values are expressed as mean ± standard error of the mean. ^‡^ indicates proportion relative to WBC.
