# Supplementary material for: Omega 3 supplementation reduces C-reactive protein, prostaglandin E2 and the granulocyte/lymphocyte ratio in heavy smokers: An open-label randomized crossover trial
Source: Front Nutr. 2022 Dec 1;9:1051418. doi: 10.3389/fnut.2022.1051418 (PMC9751896; doi:10.3389/fnut.2022.1051418)
Supplement: Supplementary file 7 [file Table_7.DOCX]

**Supplementary Table 7. The effect of omega 3 supplementation on Immune Profile.**

|  | **Active (n = 39)** | | | | **Control (n =19)** | | | |
| --- | --- | --- | --- | --- | --- | --- | --- | --- |
|  | **Baseline** | **1 mo** | **3 mo** | **6 mo** | **Baseline** | **1 mo** | **3 mo** | **6 mo** |
| **% CD45** |  |  |  |  |  |  |  |  |
| CD14 | 12.7 ± 0.8 | 13.4 ± 0.7 | 14.2 ± 0.8 | 12.2 ± 0.9 | 13.2 ± 1.0 | 14.1 ± 0.9 | 15.0 ± 1.0 | 13.5 ± 0.9 |
| CD14CD16 | 1.7 ± 0.2 | 1.7 ± 0.1 | 1.6 ± 0.1 | 1.5 ± 0.2 | 1.8 ± 0.2 | 2.0 ± 0.3 | 1.6 ± 0.2 | 1.6 ± 0.2 |
| CD14CD16- | 11.5 ± 0.7 | 12.1 ± 0.7 | 12.8 ± 0.8 | 10.9 ± 0.8 | 12.3 ± 1.6 | 13.2 ± 1.3 | 13.7 ± 1.4 | 11.1 ± 0.9 |
| CD56 bright | 0.6 ± 0.1 | 0.5 ± 0.0 | 0.5 ± 0.0 | 0.6 ± 0.1 | 0.5 ± 0.1 | 0.5 ± 0.1 | 0.5 ± 0.1 | 0.7 ± 0.1 |
| CD56 dim | 11.5 ± 0.8 | 10.2 ± 0.8^*^ | 9.7 ± 0.7^*^ | 10.6 ± 0.7 | 11.7 ± 1.3 | 11.4 ± 1.2 | 10.1 ± 1.2^*^ | 12.7 ± 1.4 |
| CD56 total | 12.1 ± 0.8 | 10.8 ± 0.8^*^ | 10.3 ± 0.7^*^ | 11.2 ± 0.7 | 12.2 ± 1.3 | 11.9 ± 1.2 | 10.2 ± 1.2 | 13.4 ± 1.4 |
| CD3 | 61.7 ± 1.6 | 62.2 ± 1.3 | 60.7 ± 1.4 | 62.7 ± 1.5 | 62.7 ± 2.0 | 61.3 ± 1.9 | 60.8 ± 2.3 | 61.0 ± 2.5 |
| CD4 | 69.6 ± 2.5 | 69.7 ± 2.4 | 70.5 ± 2.4 | 69.9 ± 2.4 | 72.6 ± 3.2 | 71.9 ± 3.3 | 70.8 ± 3.1 | 70.2 ± 3.9^*^ |
| CD4CD8 | 1.8 ± 0.3 | 1.6 ± 0.2 | 1.7 ± 0.2 | 1.6 ± 0.2 | 1.5 ± 0.2 | 1.5 ± 0.2 | 1.6 ± 0.3 | 1.5 ± 0.3 |
| CD8 | 25.5 ± 2.1 | 25.5 ± 2.1 | 25.1 ± 2.2 | 25.5 ± 2.0 | 22.8 ± 2.6 | 23.7 ± 2.9 | 23.3 ± 2.2 | 24.7 ± 3.2^*^ |
| **Treg (%CD4)** |  |  |  |  |  |  |  |  |
|  |  |  |  |  |  |  |  |  |
| FoxP3+CD25+CD127 low | 4.9 ± 0.2 | 4.3 ± 0.2^*^ | 4.4 ± 0.2 | 4.7 ± 0.3 | 3.8 ± 0.2 | 4.1 ± 0.3 | 4.1 ± 0.3 | 5.2 ± 0.3^*^ |

* denotes a significant (P < 0.05) difference when compared to baseline values. Values are expressed as mean ± standard error of the mean.
