# Supplementary material for: Omega 3 supplementation reduces C-reactive protein, prostaglandin E2 and the granulocyte/lymphocyte ratio in heavy smokers: An open-label randomized crossover trial
Source: Front Nutr. 2022 Dec 1;9:1051418. doi: 10.3389/fnut.2022.1051418 (PMC9751896; doi:10.3389/fnut.2022.1051418)
Supplement: Supplementary file 8 [file Table_8.DOCX]

**Supplementary Table 8. Summary of Blood Biomarkers in the Safety Panel.**

**Active (n = 39) Control (n = 19)**

**BL 1mo 3mo 6mo BL 1mo 3mo 6mo**

Alkaline 78.8 ± 3.8 74.5 ± 3.9^*^ 77.4 ± 4.2 72.8 ± 3.7^*^ 79.2 ± 4.9 81.5 ± 6.8 84.7 ± 10.7 82.1 ± 5.7
Phosphatase (U/L)

ALT (U/L) 22.3 ± 1.7 25.5 ± 2.6 25.1 ± 1.7 23.5 ± 1.6 21.0 ± 1.9 20.2 ± 2.5 20.7 ± 4.6 22.7 ± 2.6

AST (U/L) 24.3 ± 2.4 25.5 ± 2.3 25.6 ± 2.2 24.8 ± 2.7 22.7 ± 2.7 18.7 ± 1.1 19.0 ± 1.8 27.6 ± 4.5

Bicarbonate (mmol/L) 26.7 ± 0.4 26.2 ± 0.5 26.6 ± 0.4 26.4 ± 0.3 26.0 ± 0.6 24.9 ± 0.7 25.3 ± 1.1 27.3 ± 0.6

Bilirubin (µmol/L) 8.1 ± 0.4 9.1 ± 0.7 8.4 ± 0.7 8.5 ± 0.6 7.5 ± 0.5 7.0 ± 0.8 7.2 ± 1.1 8.3 ± 0.8

Chloride (mmol/L) 102.1 ± 0.7 101.5 ± 0.6 102.2 ± 0.6 102.5 ± 0.6 102.0 ± 1.0 102.4 ± 0.9 102.3 ± 1.0 101.8 ± 0.8

Creatinine (µmol/L) 75.6 ± 2.3 75.2 ± 3.1 76.7 ± 2.4 82.2 ± 2.5 72.7 ± 3.7 72.3 ± 3.9 80.0 ± 7.0 72.9 ± 3.6

eGFR (mL/min/1.73sqm) 79.6 ± 2.1 78.4 ± 2.3 78.6 ± 2.3 73.1 ± 2.1^*^ 82.6 ± 3.3 82.5 ± 3.4 71.8 ± 8.3 82.0 ± 3.1

Potassium ion (mmol/L) 4.3 ± 0.1 4.3 ± 0.1 4.4 ± 0.1 4.5 ± 0.1 4.4 ± 0.1 4.4 ± 0.1 4.5 ± 0.1 4.4 ± 0.1

Sodium ion (mmol/L) 140.0 ± 0.4 139.8 ± 0.4 140.5 ± 0.4 140.2 ± 0.3 140.3 ± 0.8 141.1 ± 0.7 140.8 ± 0.5 139.6 ± 0.5

Urea (mmol/L) 5.7 ± 0.3 5.6 ± 0.2 5.6 ± 0.3 5.9 ± 0.3 5.3 ± 0.4 5.7 ± 0.4 6.3 ± 0.4 5.3 ± 0.4

* denotes a significant (P < 0.05) difference when compared to baseline values. Values are expressed as mean ± standard error of the mean.
