# Supplementary material for: Omega 3 supplementation reduces C-reactive protein, prostaglandin E2 and the granulocyte/lymphocyte ratio in heavy smokers: An open-label randomized crossover trial
Source: Front Nutr. 2022 Dec 1;9:1051418. doi: 10.3389/fnut.2022.1051418 (PMC9751896; doi:10.3389/fnut.2022.1051418)
Supplement: Supplementary file 9 [file Table_9.DOCX]

**Supplemental Table 9. Reported Adverse Events in the Control and Omega-3 Arms (n=39).**

Active Control

1 mo 3 mo 6 mo 1 mo 3 mo 6 mo

Stomach issues

grade 1 2/39 1/39 2/39 0/39 0/39 0/39

grade 3 0/39 1/39 1/39 0/39 0/39 0/39

Flatulence

grade 1 8/39 3/39 8/39 0/39 0/39 0/39

grade 2 0/39 1/39 0/39 0/39 0/39 0/39

Loose stool

grade 1 7/39 5/39 5/39 0/39 0/39 0/39

grade 2 0/39 1/39 0/39 0/39 0/39 1/39

grade 3 1/39 1/39 1/39 0/39 1/39 1/39

Indigestion grade 1 1/39 2/39 1/39 0/39 0/39 0/39

Cramping 2/39 1/39 0/39 0/39 0/39 0/39

Constipation 0/39 3/39 1/39 0/39 0/39 1/39

Body ache

grade 1 1/39 0/39 0/39 1/39 0/39 0/39

grade 3 0/39 0/39 0/39 0/39 0/39 1/39

Bloating 0/39 1/39 1/39 0/39 0/39 0/39

Brittle Nail 0/39 0/39 0/39 1/39 1/39 1/39

Other adverse events Cough Asthma Death*

Pimple

Nausea

Acid reflux

Greasy hair

*Died due to surgery complications, 6 months after completion of omega-3 arm
