# Supplementary figures and images for: Omega 3 supplementation reduces C-reactive protein, prostaglandin E2 and the granulocyte/lymphocyte ratio in heavy smokers: An open-label randomized crossover trial
Source: Front Nutr. 2022 Dec 1;9:1051418. doi: 10.3389/fnut.2022.1051418 (PMC9751896; doi:10.3389/fnut.2022.1051418)

## Slide 1
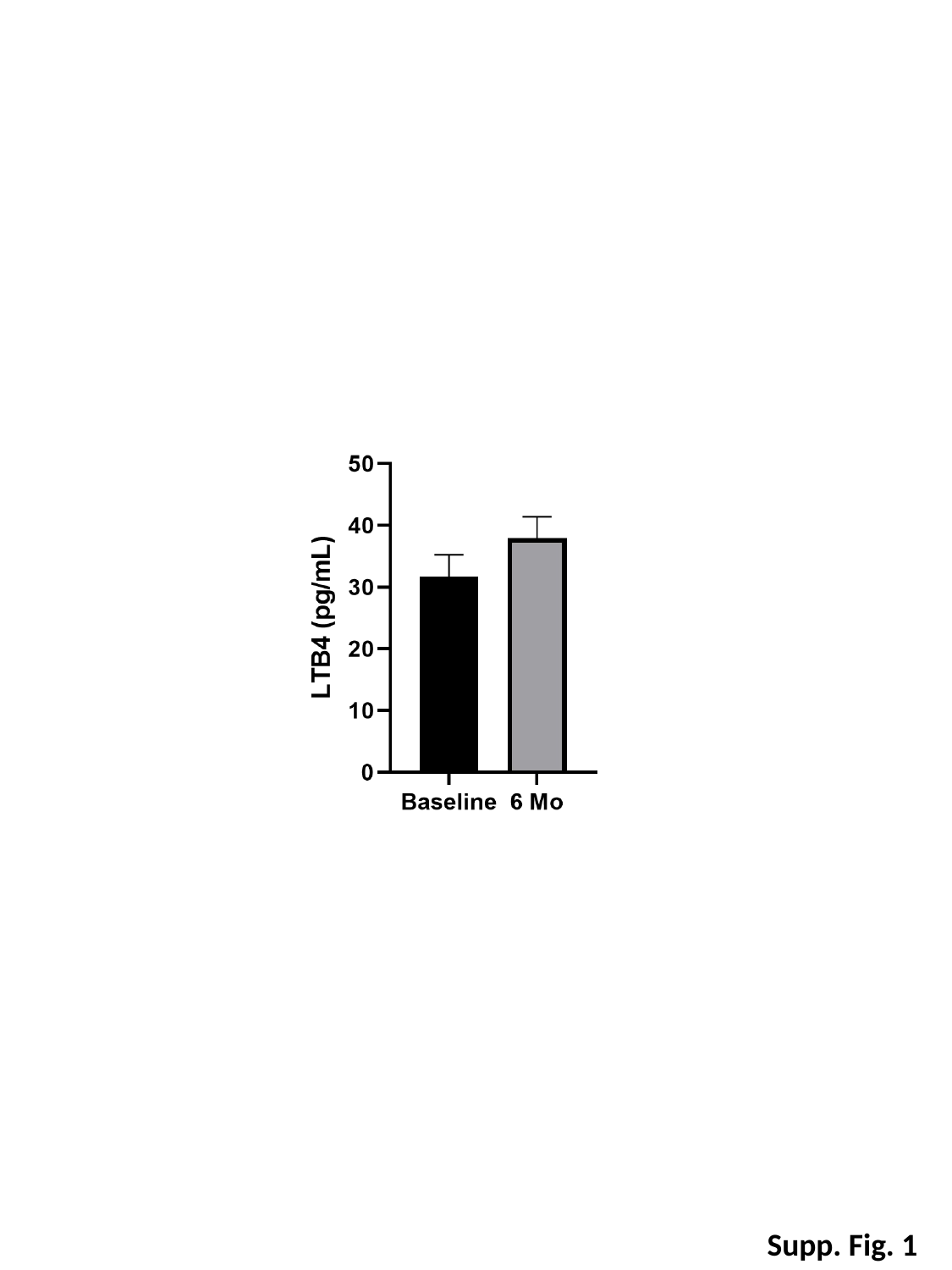

Supp. Fig. 1

Supplement: Supplementary file 10 [file Presentation_1.PPTX]
